# Predictors and Diagnostic Significance of the Adenosine Related Side Effects on Myocardial Perfusion SPECT/CT Imaging

**DOI:** 10.4274/mirt.85057

**Published:** 2014-10-05

**Authors:** Nilüfer Yıldırım Poyraz, Elif Özdemir, Barış Mustafa Poyraz, Zuhal Kandemir, Mutlay Keskin, Şeyda Türkölmez

**Affiliations:** 1 Ankara Atatürk Training and Research Hospital, Clinic of Nuclear Medicine, Ankara, Turkey; 2 Private TOBB ETÜ Hospital, Clinic of Chest Disease, Ankara, Turkey

**Keywords:** Adenosine, myocardial perfusion imaging, side effects, single-photon emission computerized tomography, computed tomography, X-ray

## Abstract

**Objective:** The aim of this study was to investigate the relationship between patient characteristics and adenosine-related side-effects during stress myocard perfusion imaging (MPI). The effect of presence of adenosine-related side-effects on the diagnostic value of MPI with integrated SPECT/CT system for coronary artery disease (CAD), was also assessed in this study.

**Methods:** Total of 281 patients (109 M, 172 F; mean age:62.6±10) who underwent standard adenosine stress protocol for MPI, were included in this study. All symptoms during adenosine infusion were scored according to the severity and duration. For the estimation of diagnostic value of adenosine MPI with integrated SPECT/CT system, coronary angiography (CAG) or clinical follow-up were used as gold standard.

**Results:** Total of 173 patients (61.6%) experienced adenosine-related side-effects (group 1); flushing, dyspnea, and chest pain were the most common. Other 108 patients completed pharmacologic stress (PS) test without any side-effects (group 2). Test tolerability were similar in the patients with cardiovascular or airway disease to others, however dyspnea were observed significantly more common in patients with mild airway disease. Body mass index (BMI) ≥30 kg/m2 and age ≤45 years were independent predictors of side-effects. The diagnostic value of MPI was similar in both groups. Sensitivity of adenosine MPI SPECT/CT was calculated to be 86%, specificity was 94% and diagnostic accuracy was 92% for diagnosis of CAD.

**Conclusion:** Adenosine MPI is a feasible and well tolerated method in patients who are not suitable for exercise stress test as well as patients with cardiopulmonary disease. However age ≤45 years and BMI ≥30 kg/m2 are the positive predictors of adenosine-related side-effects, the diagnostic value of adenosine MPI SPECT/CT is not affected by the presence of adenosine related side-effects.

## Abstract (tr)

**Amaç:** Bu çalışmada amaç yaş, cinsiyet, vücut kitle indeksi ve mevcut kardiyopulmoner hastalıklar gibi hastaya bağlı faktörlerin miyokard perfüzyon sintigrafisin de (MPS) farmakolojik stres ajanı olarak kullanılan i.v. adenozinin yan etki sıklığı üzerine etkisini araştırmaktı. Ayrıca yan etki varlığının MPS’nin koroner arter hastalığını (KAH) belirlemedeki tanısal değeri üzerine etkisi de incelendi.

**Yöntem:** Toplam 281 hastaya (109 E, 172 K; ortalama yaş: 62,6±10) entegre SPEKT/BT sistemi ile adenozin MPS uygulandı. Standart adenozin protokolü sırasında gözlenen hemodinamik yanıt ve yan etkiler belirlendi. Koroner anjiografi ve klinik takip bulguları, MPS bulgularını değerlendirmek için altın standart olarak kullanıldı.

**Bulgular:** Farmakolojik stres protokolü sırasında toplam 173 hastada (%61,6) adenozine bağlı yan etki gözlendi (grup 1); en sık görülen yan etkiler sırasıyla; ciltte kızarma, nefes darlığı ve göğüs ağrısı şeklindeydi. Diğer 108 hastada yan etki gözlenmeden test tamamlandı (grup 2). MPS’nin KAH belirlemede tanısal değeri her iki grup için benzerdi ve MPS SPEKT/BT’nin duyarlılığı %86, özgüllüğü %94 ve tanısal doğruluğu %92 olarak hesaplandı. Hava yolu hastalığı olanlarda daha sık gözlenen nefes darlığı dışında kardiyovasküler ve pulmoner hastalığı olanlarda test toleransı açısından diğer hastalardan belirgin farklılık gözlenmedi. Vücut kitle indeksi (VKİ) ≥30 kg/m2 ve yaş ≤45; yan etki sıklığını belirlemede bağımsız faktörler olarak bulundu.

**Sonuç:** Adenozin MPS; kardiyovasküler ve pulmoner hastalığı olanlar da dahil olmak üzere egzersiz yapması uygun olmayan hastalarda KAH’ını araştırmada uygun ve güvenli bir yöntemdir. Yaş ≤45, VKİ ≥30 kg/m2 olması yan etki sıklığını arttıran bağımsız faktörler olup yanetki varlığı SPEKT/BT sisteminde MPS’nin tanısal değerini etkilemez.

## INTRODUCTION

Stress gated single photon emission computed tomography myocardial perfusion imaging (SPECT MPI) is a validated, noninvasive method for evaluating patients with known or suspected coronary artery disease (CAD) (1). With recent advances in imaging technology, such as integrated SPECT/computerized tomography system (SPECT/CT), the diagnostic accuracy of MPI has also increased (2,3). The preferred stress test for MPI is physicalexercise, however pharmacologic stress (PS) agents are indicated for the patients who are not suitable for exercise testing. With a comparable sensitivity and accuracy with exercise stress test, adenosine is a potent vasodilator with a short half-life (less than 10 sec), which makes it easy to control in a clinical situation (4,5,6). On the other hand, some side-effects are observed with adenosine because of nonselective activation of adenosine receptor subtypes (5,6). Furthermore, in the presence of cardiovascular or bronchopulmonary diseases adenosine-induced changes might have some variability (6,7,8). Selective A2A adenosine receptor agonists can provide adequate coronary vasodilation for MPI with fewer side-effects as well as simplify the MPI procedure through availability to a wider range of patients and easier administrative requirements (9,10). However they are not currently available all over the world and adenosine is still valuable as a PS agent for MPI. This study is set out with the aim of investigation the relationship between adenosine-induced changes and patient characteristics such as age, gender, body mass index (BMI), cardiovascular disease (CVD) or mild airway disease (MAD) in a Turkish cohort. The second goal of the current study was to evaluate the effect of the presence of adenosine-related side-effects on the diagnostic value of MPI with integrated SPECT/CT system. 

## MATERIAL AND METHODS

**Patients**

A total of 302 patients were referred to our nuclear medicine clinic for MPI with PS between December 2010 and August 2012. Hypotensive (systolic blood pressure <90 mmHg) patients and patients with evidence of advanced atrioventricular (AV) block, acute myocardial infarction (MI) or unstable angina pectoris within 1 week, severe bronchoconstructive or bronchospastic lung disease (forced expiratory volume in 1 second (FEV1) <50%) were excluded from PS test with adenosine, however 48 patients with MAD were included in the study (11). Patients with MAD were pretreated with 2 puffs of salbutamol (inhaled beta2-adrenergic agonist) before adenosine infusion protocol. Total of 281 patients were enrolled in this prospective study. Table 1 lists the characteristics of the patients.

All patients were asked to avoid caffeine-containing products for at least 24 h before the test, and were instructed to stop calcium antagonists before 24 h, β-blockers before an interval equivalent to 5 half-lives and nitrate preparations were witheld on the day of the adenosine MPI study.

**Pharmacologic Stress Protocol**

Adenosine (adenosine-L.M. i.v., Abfenfarma) was infused at 140 µg/kg per minute intravenously, using an accurate infusion pump over 6 minutes. After 3 minutes of infusion the radionuclide perfusion tracer (Tc-99m methoxyizobutylizonitrile: MIBI or Thallium-201: Tl-201) was administered intravenously and the infusion was continued for an additional 3 minutes. According to the the severity of the side-effects, adenosine dose was decreased to 100 µg/kg/min or the infusion was terminated after the radiotracer was injected. If the symptoms persisted, in spite of termination of infusion, aminophylline -an adenosine receptor antagonist- was administered at 75 mg over 1 min to a maximal dose of 225 mg.

Patients were monitored and a 12-lead electrocardiogram (ECG) was recorded and blood pressure (BP) and heart rate (HR) were recorded every 2 minutes during stress procedure. All symptoms during and after termination of adenosine infusion were recorded in a standart form with scoring according to the severity and duration (1:mild, 4:serious). Symptoms with scores of 2, 3, 4 were considered to be positive. The severity of side-effects was also categorized and recorded as; spontaneously tolerated, tolerated with dose adjustment, required termination of adenosine infusion or required reversal with aminophylline.

**Myocardial Perfusion Imaging Protocol**

One day stress/rest gated MPI protocol was applied to all patients. Tc-99m MIBI was the perfusion agent used in the majority of patients (87%), but 13% received Tl-201. MPIs were performed on an integrated SPECT/CT system (Infinia Hawkeye 4-GE Healthcare) which combines a low-dose multislice CT with a dual-head camera in a single gantry. SPECT was done using a low-energy, high-resolution, parallel hole collimator and with the following parameters: a 20% symmetric window at 140 keV; a 64x 64 matrix; 25 seconds/frame and gating included 16 frames/cycle. For the attenuation map, single-slice nonspiral CT (x-ray tube current, 2.5 mA; voltage, 140 kVp) with a slice thickness of 10 mm and a scan time of more than 5 min for a typical 13-cm field of view was obtained without intravenous contrast material and without a breath-hold.

After reconstruction AC maps were generated as previously reported in detail (12). Data were thereafter quantitatively analyzed on the commercially available QPS/QGS software version 4.0 (Cedars-Sinai Medical Center) (13). A 20-segment model was used to determine percentage uptake of the radionuclide. The software generates a polar-map from myocardial SPECT images, then mean % tracer uptake in each segment is automatically scored from normal (0) to absent (4) using a 5-point model. Subsequently, summed stress scores (SSS), summed rest scores, and summed difference scores (SDS) were calculated from the 20 segment scores. An SSS of ≥4 and SDS of ≥2 were considered abnormal (12,13). At the same time, wall motion, and wall thickening were incorporated with both the extent and severity of perfusion defects. A reversible perfusion defect was defined as SSS≥4 which was associated with a SRS≤2. Reversible perfusion defects were considered to be myocardial ischemia, whereas fixed perfusion defects (SSS≥4 and SDS≤2 ) with concurrent regional wall motion abnormalities were considered to be myocardial scar. The location of the perfusion defects were de¬scribed in regard to the left ventricular wall and the coronary vascular territory, while defects in more than one region associated with a particular coronary artery were considered to demonstrate multivessel disease. Left ventricular dilation on stress images despite with rest dilation were considered to have transient ischemic dilation.

**Follow-up and Final Diagnosis**

A total of 102 patients had CAG (within last 3 months before MPI or after MPI study) and other 179 patients clinically followed-up for at least 6 months for cardiac events. CAD with CAG was defined as the presence of at least one of the three major epicardial coronary arteries exhibited luminal narrowing 50% or greater. Furthermore, a total of 32 patients designated as severe CVD group which consisted of patients with prior MI, multivessel disease or congestive heart failure (CHF) with left ventricular ejection fraction (LVEF) <50% according to clinical, laboratory and MPI findings. For the estimation of diagnostic value of adenosine MPI with integrated SPECT/CT system, coronary angiography (CAG) or clinical follow-up were used as gold standard.

**Statistical Analysis**

Continuous variables are expressed as mean ± standard deviation. Categorical variables are expressed as counts and percentages. Differences in continuous variables were assessed by independent student’s t-test and qualitative data were compared using the Pearson’s chi- square test. A probability value of p<0.05 was considered significant and two-tailed p values were used for all statistics. We used binary logistic regression analysis, the odds ratios (OR’s) and 95% confidence intervals (CI) to identify potential predictors of adenosine-related side effects. Optimal cut-off values of age and BMI for identifying the risk of adenosine-related side-effects were determined using receiver operating characteristic (ROC) analysis. Statistical analyses were performed using SPSS (statistical package for social science) software version 11.5. 

This prospective study is approved by university research ethics committee (Yıldırım Beyazıt University Research Ethics Committee, Ankara, Turkey) and written informed consents were obtained from all patients.

## RESULTS

There were no deaths or myocardial infarction in our study group during or after adenosine MPI. Standard protocol was completed in 227 patients (80%), dose reduction was required in 31 pts (11%), and infusion was terminated early in 23 pts (8%). The reasons for the early termination or dose reduction were marked ECG changes (ST depression >2 mm, second degree AV block, significant arrhythmia), chest pain or severe dyspnea (wheezing). Aminophylline was only given to 3 patients (1%) who had angina with ST depression (n=1) or third degree AV block (n=2). The typical hemodynamic response to adenosine infusion was a slight reduction in BP (mean 12.9±10.2 mmHg for systolic blood pressure) with a compensatory increase in HR (mean 12.6±8.7 beat per minute). In general, adenosine related hemodynamic changes, more specifically for HR were reduced with aging and in patients with diabetes mellitus (DM) or severe CVD, however the differences were not statistically significant. On the other hand, side-effects of adenosine were observed significantly more common in patients with marked hemodynamic changes (decrease in SBP >20 mmHg) when compared to patients with modest hemodynamic response or without change (77.6% vs. 53.5%, p<0.001).

**Side-effects of Adenosine**

A total of 173 patients (61.6%) experienced one or more side-effects of adenosine during PS (group 1); the common side-effects were flushing, dyspnea, chest pain, gastrointestinal discomfort (GID), headache, throat-neck discomfort (TND), and ECG changes (arrhythmia or ST segment depression). The side-effects of adenosine are summarized in Table 2. Other 108 patients of the study group (38.3%) completed PS test without any side-effects (group 2). Patient characteristics of group 1 and 2 were summarized in Table 3. Mean age of group 1 was significantly lower than group 2. On the other hand, mean weight and more specifically mean BMI were significantly higher in group 1 than group 2. According to the gender; more side effects were observed in women than in men; however, the difference between the totalfrequency of all side-effects was not statistically significant. While flushing, dyspnea, chest pain, and headache were more commonly observed in women, gastrointestinal discomfort, TND and ECG changes were more commonly observed in men.

Thirty three of 48 patients with MAD were in group 1 and adenosine-related side-effects were observed more commonly in patients with MAD (68.8%) than the others (60.5%). However the differences between the frequencies of adenosine related side-effects were not statistically significant, except for dyspnea (39.6% vs. 24%, p=0.027). Dose reduction was needed in 6 patients, infusion was stopped early in only 4 patients and aminophylline was not required in any case with MAD. Bronchospasm manifested as wheezing was observed in only 6 (12.5%) of those patients and resolved in 2 patients with dose reduction and in 4 patients with discontinuing infusion. 

Total frequencies of all side-effects were lower in diabetic patients or patients with severe CVD than others, however the differences were not statistically significant. 

**Figure 1:**
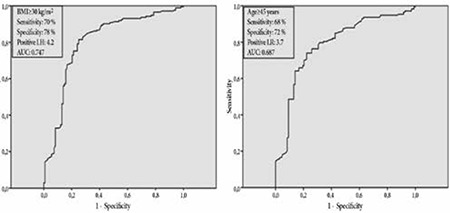
Receiver-operator characteristic analyses to define cut-off valuesfor body mass index and age that are predictors of adenosine-related side-effects.

Regression analysis showed that BMI and age were independent predictors of adenosine-related side-effects. OR (95% CI) for BMI was 3.2 (1.3-6.7) and for age was 0.77 (0.62-0.97) (p=0.001). ROC analysis revealed that a BMI cut-off ≥30 kg/m2 and an age cut-off≤45 years had 70%, 68% sensitivity and 78%, 72% specificity, respectively (Figure 1).

**Myocardial Perfusion Imaging Findings and Follow-up**

MPI findings were considered as normal in other 197 patients, attenuation correction (AC) with CT decreased the rates of probably normal results especially in obese patients with high soft tissue attenuation. On the other hand fifty one patients in group 1 and 33 patients in group 2; total of 84 patients were found to have perfusion defects in a total 116 vascular territories (single vessel in 57 patients and multivessel in 27 patients) with stress MPI SPECT/CT. In rest images; defects were reversible in 60 of 116 vascular territories, while in 56 of 116 vascular territories perfusion defects were fixed. According to the findings of MPI, 24 patients had fixed perfusion defect (myocardial scar), 14 patients had both reversible hypoperfusion with fixed perfusion defect (ischemia and myocardial scar) and 46 patients had only reversible hypoperfusion (myocardial ischemia).

Of those 84 patients with abnormal MPI findings, 70 patients had a coronary angiogram and 12 patients had no significant coronary occlusion (false positive MPI), however there was no need of CAG for 14 patients with known prior MI and fixed perfusion defect in MPI study.

On the other hand, 32 of 197 patients with negative MPI study underwent CAG within 3 months, based on clinical criteria and 11 patients had coronary occlusion in (false negative MPI). It was important to note that one patient with balanced three-vessel stenosis was missed by MPI, other patients had single vessel disease.

As a result, MPI findings were correlated with CAG in 102 patients and with clinical follow-up in 179 patients for cardiovascular events; sensitivity of adenosine MPI with integrated SPECT/CT system was calculated to be 85%, specificity was 94% and diagnostic accuracy was 92%. Table 4 summarized the distribution of true positive, true negative, false positive and false negative cases among groups 1 and 2. 

## DISCUSSION

Our results corroborated the previous studies which indicate that adenosine is a well tolerated PS agent in patients who are not suitable for exercise stress test as well as patients with cardiovascular disease or MAD. However dyspnea was commonly seen, bronchospasmmanifested as wheezingwas observed in only 6 of 48 patients with MAD in the present study. These results are consistent with earlier findings which shows that dyspnea is not correlated with impaired respiratory resistance in patients with MAD, and is most likely secondary to stimulation of vagal C fibers in the lungs (14,15,16,17). The main goal of the current study was to investigate the relationship between adenosine-induced changes and patient characteristics in a Turkish cohort. Our findings are consistent with previous observation that obese or young patients more suffered from side-effects of adenosine and suggest that BMI ≥30 kg/m2 and age ≤45 years are independent predictors for adenosine-related side-effects in PS. It is known that side-effects of adenosine are dose-dependent and overweight patients are exposed to higher dose of adenosine during PS (6,7). On the other hand, for weight-height indices, the BMI provides more accurate estimates of body composition than BW, because its correlation with body fat percentage is high, and its correlation with body height is low (18). Our study group consisted of patients who were not suitable for exercise testing and were mostly overweight, however body weight was not accordant with BMI in some cases, more specifically in female patients. Therefore, we have taken into account the values of BMI instead of weight to determine cut-off values of predicting factors. In addition, men tolerated adenosine better than women in the current study, possibly explained by a relatively greater volume of distribution of the infused vasodilator in women compared with men attributable to higher fat-to muscle ratio (19). The age-dependent results of hemodynamic response and side-effects of adenosine have been described earlier, the findings of the current study are in agreement with those studies that the hemodynamic response to adenosine were reduced with aging and side-effects of adenosine were observed significantly more common in patients with marked hemodynamic changes (6,7,8,20). Similar with elderly patients, patients with DM had reduced hemodynamic response to adenosine and experienced side-effects less frequently in the current study, possibly attributable to autonomic dysfunction. Our findings are also consistent with the known association of decreased vasodilation response with severe CVD such as prior MI or CHF, that is because of the increased sympathetic activity (6,7). Karamitsos et al. (21) have identified an age cut-off (≥ 65 years) for inadequate hemodynamic response to i.v. adenosine which was used for cardiovascular magnetic resonance perfusion studies and concluded that inadequate peripheral vascular response to adenosine might indicate reduced coronary vasodilation. Carlsson et al. (22) have recently reported that decreased hemodynamic response and hyperemia during adenosine was lower in patients with shorter caffeine abstinence compared to 24 hour, however all patients were asked to avoid caffeine-containing products for at least 24 h before the test in the current study. Similar differences in adenosine responsiveness have also been reported in MPI studies, however it was reported that diagnostic accuracy of MPI for detecting CAD was not affected by the presence or lack of significant changes in HR and blood pressure in response to adenosine (23,24). Current findings corroborate the latter study and suggest that presence of side-effects due to changes in hemodynamic response should not be used to assess the effectiveness of adenosine stress during MPI study. In the present study, the sensitivity, specificity and diagnostic accuracy of MPI SPECT/CT in patients who experienced adenosine related side-effects were similar with others. However the diagnostic values were slightly higher than the values of standard MPI SPECT. This result may be explained with the integrated SPECT/CT system which provides accurate CT based attenuation correction especially in overweight and obese patients and increased specificity and diagnostic accuracy of MPI (2,3).

According to the previous findings and additional evidence of the present study, modified adenosine protocols with dose reduction or short duration of infusion might be preferred in obese (BMI ≥30 kg/m2) and young (age ≤45 years) patients to avoid discomfortable side-effects of adenosine. A 4-minute protocol proposed by O’Keefe et al. was found to be equally effective for the detection of CAD compared to the standard 6 minutes protocol (14). Low-level exercise might also be performed in combination with pharmacologic stress but it is not recommended in patients with LBBB or for patients with pacemaker (19,25).

**Study Limitations**

The major limitation of this single-center study is that the results are based on a population referred for MPI and may not be applicable to a broader population. Although all data were collected and entered prospectively, the study is retrospective. Another limitation is that the airway disease group is small and the bronchoconstrictive changes in those patients could not be evaluated by respiratory function tests during the PS.

## CONCLUSION

Adenosine MPI is a feasible and well tolerated method in patients who are not suitable for exercise stress test as well as patients with cardiopulmonary disease. However age ≤45 years and BMI ≥30 kg/m2 are the positive predictors of adenosine-related side-effects, the diagnostic value of adenosine MPI study with integrated SPECT/CT is not affected by the presence of adenosine related side-effects or hemodynamic changes.

**Conflicts of Interest**

There are no conflicts of interest.

## Figures and Tables

**Table 1:**
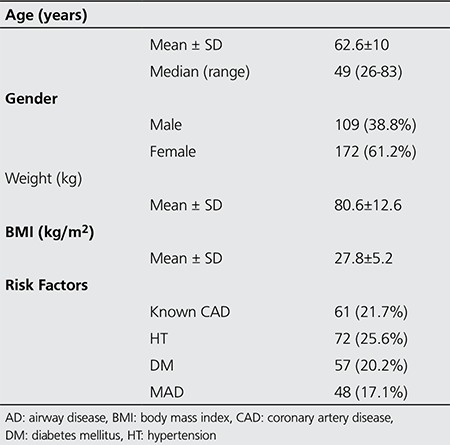
Characteristics of the patient group (n=281)

**Table 2:**
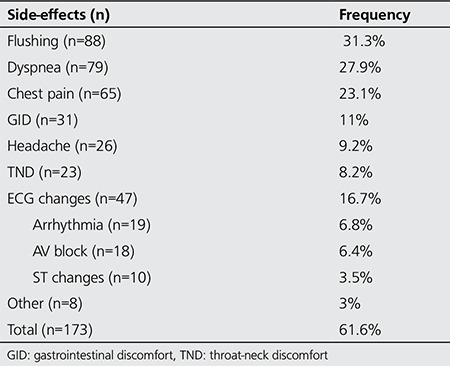
The side-effects profile and their frequenciesduring adenosine stress test for myocardial perfusion imaging

**Table 3:**
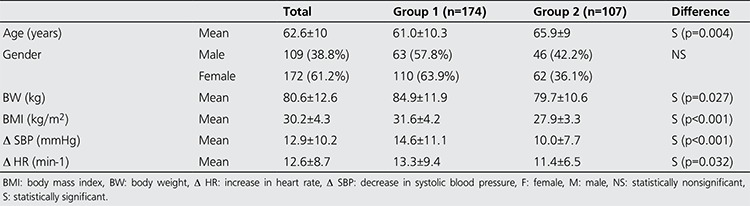
Characteristics of the patients with or without side-effects (n=281)

**Table 4:**
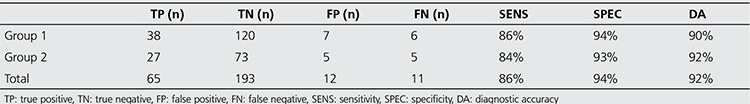
Distribution of true positive, true negative, false positive and false negative MPI findings among groups 1 and 2
